# Could a DJ-1/PARK7 pathogenic variant be a cause of cardiac hypertrophy?—a case report

**DOI:** 10.1093/ehjcr/ytaf278

**Published:** 2025-06-10

**Authors:** Mehmet Emre Ozerdem, Müge Akbulut, Kerim Esenboga

**Affiliations:** Faculty of Medicine Cardiology Department, Ankara University, Altindag 06230, Turkey; Faculty of Medicine Cardiology Department, Ankara University, Altindag 06230, Turkey; Faculty of Medicine Cardiology Department, Ankara University, Altindag 06230, Turkey

**Keywords:** hypertrophic cardiomyopathy, DJ-1/PARK7, Parkinson disease, Case report

## Abstract

**Background:**

DJ-1, a protein encoded by the PARK7 gene, is crucial in the regulation of oxidative stress and mitochondrial function. Experimental studies in murine models suggest that DJ-1 deficiency results in pronounced cardiac hypertrophy and an elevated risk of heart failure, especially under conditions of oxidative stress. Nonetheless, this association had not yet been substantiated in human studies.

**Case Summary:**

A 37-year-old male with early-onset Parkinson's disease due to a pathogenic variant of *PARK7* presented with chest pain. Initial examination showed voltage criteria for left ventricular hypertrophy on ECG and concentric hypertrophy of the left ventricle on transthoracic echocardiography. Genetic testing confirmed a homozygous DJ-1 mutation. Other potential causes of concentric left ventricular hypertrophy, such as cardiac amyloidosis, Fabry disease, and sarcoidosis, were ruled out, leading to a final diagnosis of hypertrophic cardiomyopathy (HCM). A genetic analysis conducted to identify mutations in genes associated with HCM yielded negative results, supporting the conclusion that the patient’s cardiac hypertrophy may be linked to the pathogenic variant of *PARK7.*

**Discussion:**

To the best of our knowledge, this case represents the first documented instance in humans where the loss of DJ-1 protein has been implicated as a probable aetiology of HCM.

## Graphical Abstract

**Graphical Abstract:**
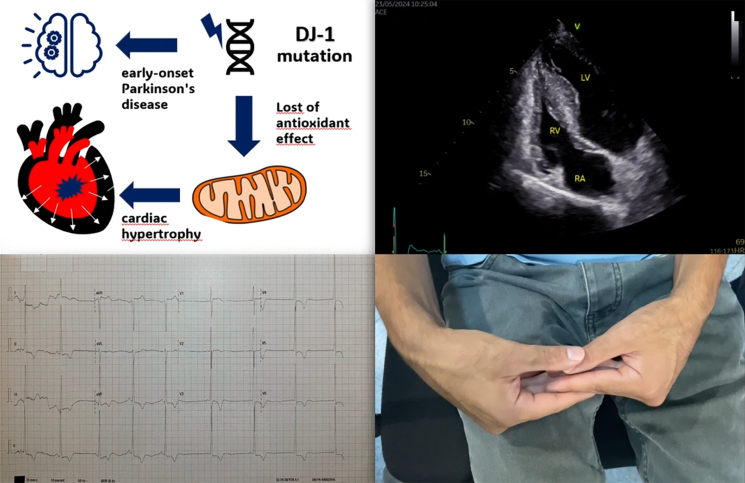

Learning points

- Pathogenic mutations in the human PARK7 gene encoding protein DJ-1 may represent an unrecognized cause of cardiac hypertrophy.
- It is essential to screen patients with early-onset parkinsonism carrying pathogenic mutations in the human PARK7 gene encoding protein DJ-1 for cardiac involvement.

## Introduction

DJ-1, also known as Parkinson’s disease protein 7 (PARK7), is a highly conserved, 189-amino-acid dimeric protein that is widely expressed across human tissues under physiological conditions.^1^ DJ-1 plays a pivotal role in the regulation of oxidative stress and mitochondrial function and has been implicated in autophagy, highlighting its broader involvement in cellular homeostasis and stress response mechanisms.^1^ Experimental studies have shown that DJ-1 deficiency in murine models leads to exaggerated cardiac hypertrophy and a predisposition to heart failure under conditions of oxidative stress.^2,3^ However, the relationship between *PARK7* mutations and cardiac hypertrophy in humans has yet to be established. In this report, we present a case of hypertrophic cardiomyopathy (HCM) identified in a patient with early-onset parkinsonism associated with a pathogenic variant of *PARK7.*

## Summary figure

**Figure:**
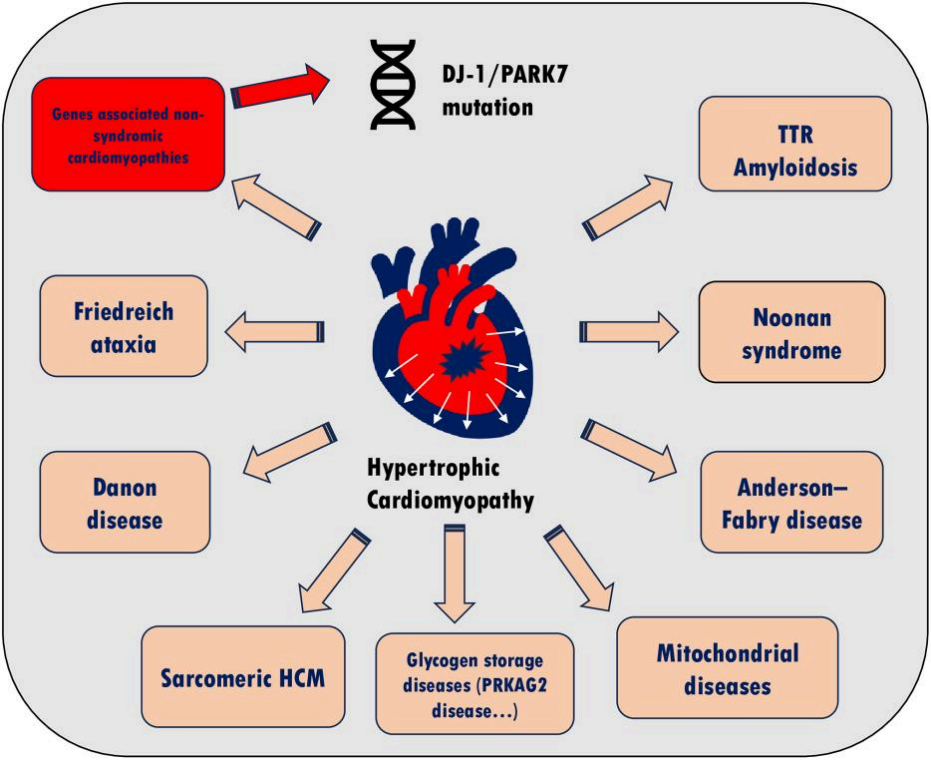


## Case presentation

**Figure 1:**
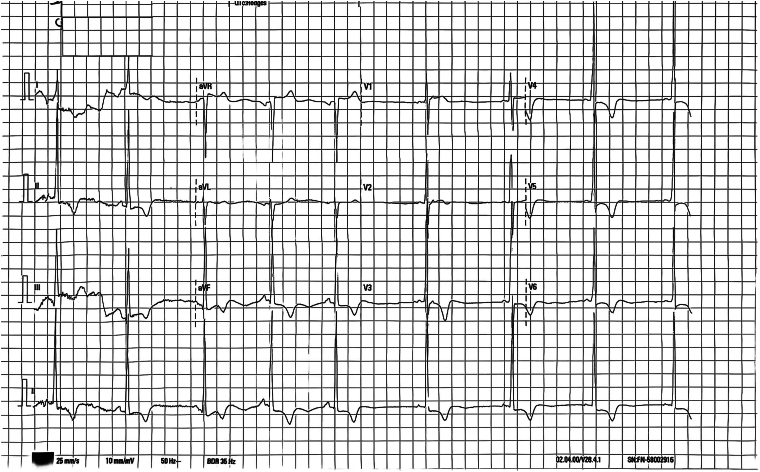
The electrocardiogram is consistent with left ventricular hypertrophy. (V_1_S + V_6_R = 40 mm) Additionally, a strain pattern associated with hypertrophy is observed.

**Figure 2:**
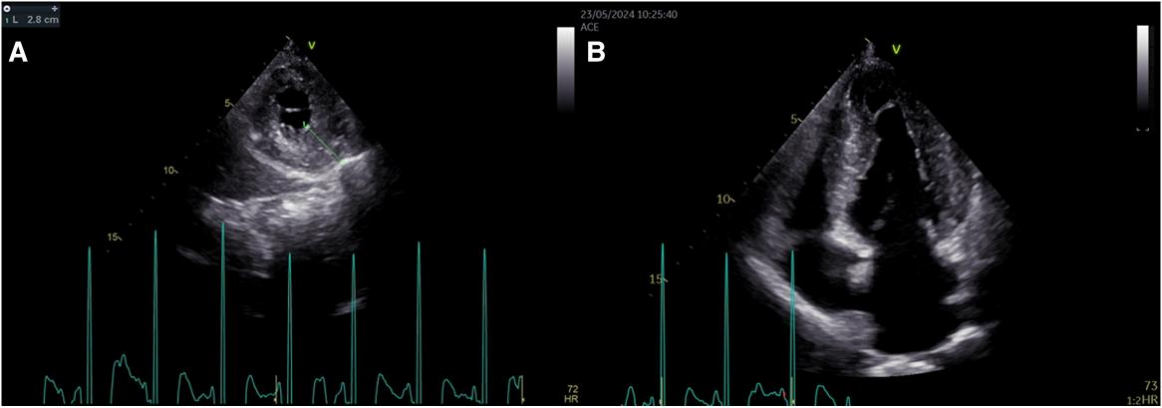
In (*A*), the parasternal short-axis view shows a maximum wall thickness of 28 mm in the apical region of the left ventricle. In (*B*), the apical four-chamber view demonstrates marked left ventricular hypertrophy.

**Figure 3:**
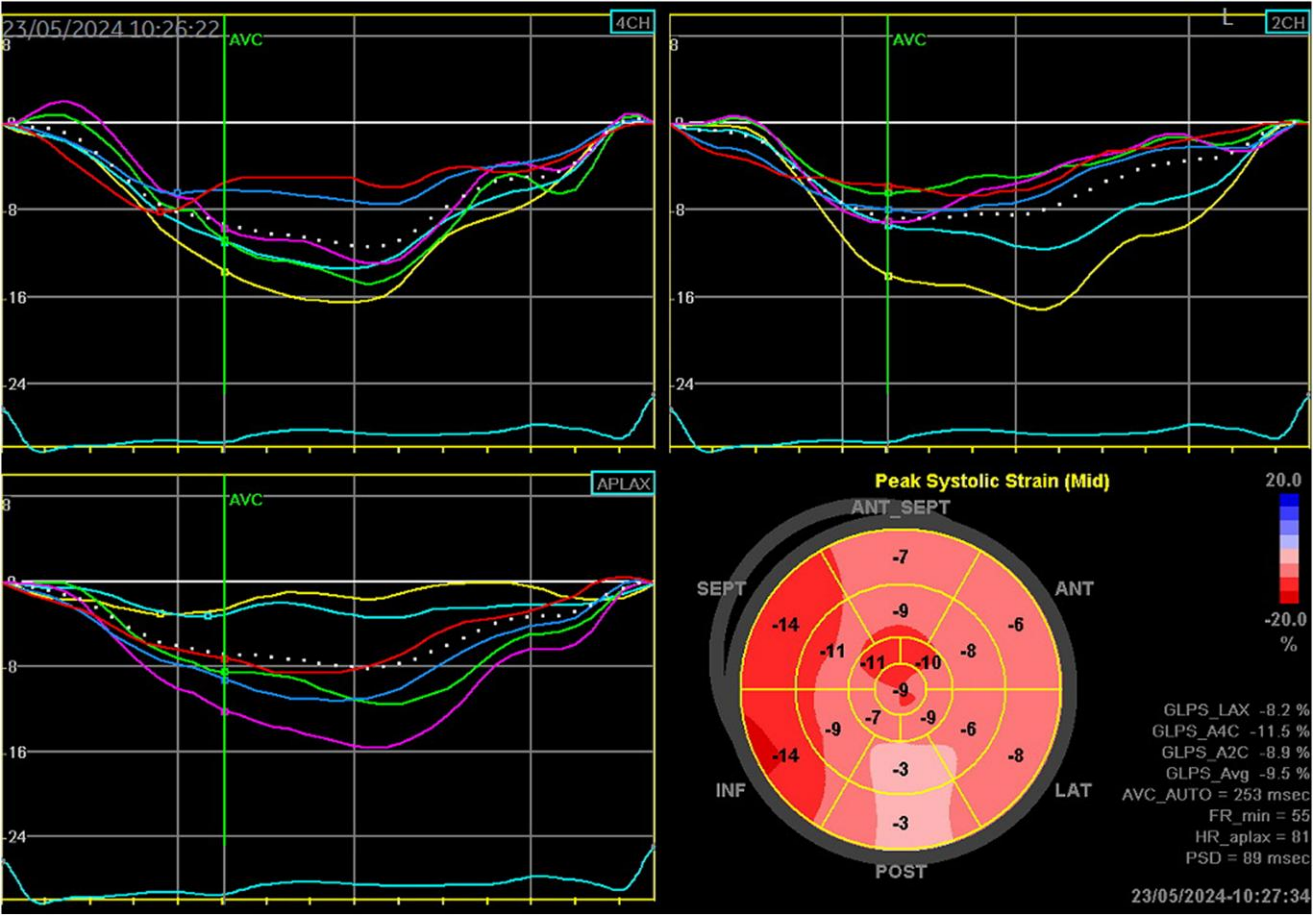
The patient’s GLS, assessed by speckle-tracking echocardiography, was calculated as −9.5%. No apical sparing pattern was observed.

A 37-year-old male patient presented to the emergency department with chest pain lasting for several days. The patient described the pain as a sudden-onset sharp pain localized to the left sternal area that worsened with body movements. He indicated that the pain alleviates with the use of non-steroidal analgesics but recurs after a few hours. He reported no sensation of burning or chest tightness. His medical history was notable for a diagnosis of early-onset Parkinson's disease (PD) at the age of 26. At that time, a genetic analysis was carried out and revealed a homozygous *DJ-1* (*PARK7; NM_00762.5 c302T* *>* *C p.(Leu101Pro)* mutation). The patient’s mother and sister were also heterozygous carriers of the mutation. The father passed away from colon cancer 8 years prior to the diagnosis of the disease; consequently, his genetic analysis could not be performed and remains unknown. The patient was started on levodopa and benserazide hydrochloride treatment for his PD. He denied any prior cardiac symptoms. The family history was also negative for any cardiac diseases and sudden deaths at a young age. Also, the patient's parents were not consanguineous. The patient had no prior history of diabetes mellitus, hypertension, or hyperlipidaemia. Also, the patient denied the use of tobacco or drugs. On physical examination, the patient's blood pressure was recorded as 120/75 mmHg, and the pulse was rhythmic. Electrocardiography (ECG) showed sinus rhythm with signs of left ventricular hypertrophy (*Figure 1*). Laboratory findings included creatinine of 0.93 mg/dL, haemoglobin of 16.1 g/dL, NT-proBNP of 672 pg/mL, and negative serial high-sensitive troponin T. The pain was evaluated as being of non-cardiac origin. However, a transthoracic echocardiography (TTE) was planned due to the elevated NT-proBNP levels. TTE revealed concentric hypertrophy of the left ventricle. The maximal wall thickness of the left ventricle was 28 mm in the apical region, resulting in the obliteration of the apex during systole. (*Figure 2*; Supplementary material online, *Videos S1* and *S2*) Pressure gradient in the left ventricular outflow tract and systolic anterior motion of the mitral valve were not evident. Left ventricular ejection fraction was 69%, and the global longitudinal strain (GLS) of the left ventricle was −9.4%, with no evidence of apical sparing (*Figure 3*). Additionally, significant hypertrophy of the right ventricular free wall was observed, with a measured thickness of 11 mm. There was no evidence of valvular pathology, and systolic pulmonary artery pressure could not be measured due to the absence of tricuspid regurgitation. The patient’s blood and urine protein electrophoresis results were within normal limits. Also, a bone scintigraphy performed for TTR-amyloidosis was negative. The patient's chest X-ray showed no evidence of hilar lymphadenopathy. Also, serum ACE levels were normal, ruling out sarcoidosis. Furthermore, the enzyme activity test for alpha-galactosidase was normal. Given all these considerations, the most likely diagnosis in this patient was HCM. Cardiac magnetic resonance imaging was planned to detect the extent of scarring to assess the risk of sudden cardiac death (SCD); however, despite multiple attempts, the MRI could not be performed due to the patient's generalized anxiety disorder associated with the pathogenic variant of *PARK7.* Additionally, to assess SCD risk, the patient was monitored with a 72-h Holter, during which no episodes of non-sustained or sustained ventricular tachycardia were observed.

Whole exome sequencing was performed to investigate potential genetic syndromes associated with HCM, yielding negative results for mutations in sarcomeric, non-sarcomeric, and PRKAG2 genes. Prior to the cardiac evaluation, a neurologist-initiated multi-gene panel analysis targeting mitochondrial disorders was performed, but the findings were inconclusive. Unfortunately, due to the unavailability of a cardiac biopsy, mitochondrial DNA analysis derived from cardiac myocytes could not be undertaken. Nevertheless, given the normal serum creatine phosphokinase and lactate levels, the clinical suspicion for a mitochondrial aetiology remained low.

**Figure 4:**
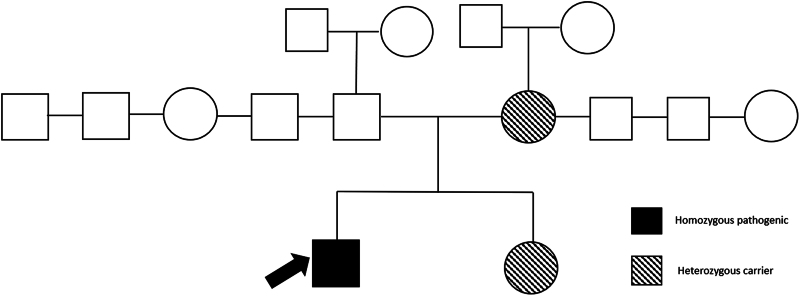
The patient's pedigree is presented. The individual indicated by an arrow represents the patient. The figure is annotated for the *DJ-1 (PARK7; NM_00762.5 c.302T* *>* *C p.(Leu101Pro))* mutation. The mother and sister were identified as heterozygous carriers.

Family screening revealed normal ECG and echocardiography results in the first-degree relatives. None of the family members exhibited the HCM phenotype. The pedigree chart is presented in *Figure 4*. Considering all these findings, and after reviewing the literature suggesting the protective effects of DJ-1 on cardiac apoptosis, we concluded that the patient’s excessive cardiac hypertrophy was possibly related to the pathogenic variant of *PARK7.* The European Society of Cardiology 5-year risk of SCD was calculated as 2.49%. Therefore, we did not plan an intracardiac defibrillator implantation for primary prevention. Metoprolol 50 mg OAD was added to the medical treatment. The patient continues to attend routine outpatient visits every 6 months. He remains asymptomatic and has not experienced any arrhythmic episodes.

## Discussion

In humans, DJ-1 was first identified in 2003 as being encoded by the *PARK7* gene, which is linked to early-onset familial forms of PD.^1^ The DJ-1 protein is neuroprotective, exerting its effects primarily through the modulation of oxidative stress and mitochondrial function. Mutations or deletions in the *PARK7* gene can result in mitochondrial dysfunction, impaired autophagy, neuroinflammation, dopamine depletion, and cell death, culminating in bioenergetic failure and subsequent development of PD.^4,5^ Given the widespread expression of DJ-1 in human tissues, mutations in the *PARK7* gene may have implications not only for neurodegenerative conditions like PD but also for other pathological states where autophagy and oxidative stress are key factors.^6^ Experimental studies have shown that DJ-1 deficiency in murine models leads to exaggerated cardiac hypertrophy, and a predisposition to heart failure under conditions of oxidative stress, though the baseline echocardiographic parameters were comparable between the DJ-1_−/−_ and DJ-1_+/+_ groups.^2,3^ Specifically, DJ-1_−/−_ mice exhibited exaggerated pathological cardiac hypertrophy, increased reactive oxygen species (ROS) production, elevated cardiomyocyte apoptosis, and heightened susceptibility to heart failure.^3^ These findings suggest that DJ-1 plays a critical protective role in the heart by maintaining the balance between ROS production and clearance.^3^ Moreover, a decreased interaction between Parkin interacting substrate (PARIS) and DJ-1 has been observed in hypertrophic cardiomyocytes. DJ-1 has been shown to regulate PGC1α transcription and mitochondrial biogenesis by inhibiting the SUMOylation of PARIS, further supporting its protective role in cardiac function under stress conditions.^7^ Despite the compelling evidence from preclinical studies, no human studies have yet investigated the role of DJ-1 in cardiac disease.

Pathological cardiac hypertrophy is relatively common in the general population, with most of the cases clinically classified as HCM. However, a broad spectrum of HCM phenocopies-including Danon disease, Fabry disease, and various metabolic and mitochondrial diseases—should be considered in the differential diagnosis. While whole genome analysis can identify many of the abovementioned conditions, ruling out certain rare diseases may require more in-depth investigations like histopathological evaluation of myocardial tissue and targeted genetic analyses, including mitochondrial DNA sequencing. In our case, common phenocopies like Fabry and Danon were excluded through appropriate diagnostic testing. However, a major limitation was the inability to obtain myocardial tissue for biopsy, which precluded both histopathological evaluation and genetic analysis of myocardial-derived mitochondrial DNA. Nevertheless, given the normal serum creatine phosphokinase and lactate levels, the clinical suspicion for a mitochondrial aetiology remained low. Also, specialized genome sequencing for inherited metabolic diseases was not performed. However, prior to our cardiovascular evaluation, the patient underwent neurological assessment for his early-onset Parkinsonism, which included a multi-gene panel analysis targeting neurological phenotypes; this yielded negative results. The absence of multi-organ involvement further diminished the likelihood of a specific inherited metabolic disease. Given all available clinical and genetic data, and following the exclusion of other potential aetiologies, DJ-1 deficiency emerges as a possible contributing factor to the observed HCM in the present case.

In conclusion, HCM is the most prevalent genetic cardiovascular disorder in the general population. Although a wide array of genes associated with HCM have been identified, there is no clinical information regarding pathological left ventricular hypertrophy associated with pathogenic variants of *PARK7.* The present case report is important as it represents the first clinical presentation of a pathophysiological mechanism previously only demonstrated in animal studies. Although the pathogenic variants of *PARK7* are primarily known as a genetic cause of early-onset PD, they can also contribute to the development of HCM. Thus, cardiac screening in patients with this mutation may be beneficial (graphical abstract). Nevertheless, considering the limitations that weaken the strength of our hypothesis, future studies are required to further elucidate the causative role of the pathogenic variants of *PARK7* in cardiac hypertrophy in humans.

## Supplementary Material

ytaf278_Supplementary_Data

## Data Availability

The authors confirm the availability of the data used in this case report.

## Lead author biography

Dr. Mehmet Emre Özerdem graduated from Ankara University Faculty of Medicine in 2019. Following his graduation, he was accepted into the cardiology residency program at his alma mater, where he is currently a fourth-year resident. His professional interests within cardiology include interventional cardiology and imaging.

## Supplementary material

**Consent:** 19 Written consent for submission and publication of this case report, including images and associated text has been obtained from the patient’s next of kin, in line with COPE guidance.

**Funding:** This research has not received any funding.
